# Effects of dietary hemp seed oil to sows on fatty acid profiles, nutritional and immune status of piglets

**DOI:** 10.1186/s40104-020-0429-3

**Published:** 2020-03-18

**Authors:** D. Vodolazska, C. Lauridsen

**Affiliations:** Department of Animal Science, Faculty of Technical Sciences, Aarhus University, Blichers Allé 20, 8830 Tjele, Denmark

**Keywords:** Arachidonic acid, Immunoglobulin, Omaga-3 fatty acids, Pig, Vitamin E

## Abstract

**Background:**

The oil from industrial hemp seeds (*Cannabis sativa*) is an ideal source of stearidonic acid, which is a precursor fatty acid for the long-chained n-3 polyunsaturated fatty acids. These fatty acids are important for neonatal development, health and immunity. Hemp seed oil has been investigated for the influence on human health, but research on the impact in pig nutrition is scarce. The aim of our research was to study the effect of dietary hemp seed oil relative to soybean oil to lactating sows on the transfer of fatty acids to the off-spring and the effect on piglets’ immune and nutritional status.

**Results:**

The fatty acid composition of the hemp seed and the soybean oil influenced the fatty acid composition of sow plasma, colostrum and mature milk. The highest proportion of C18:3n-3, C18:4n-3 and C20:4n-6 was obtained in mature milk fat of sows fed 5% hemp seed oil diet when compared to the other dietary fat sources (5% soybean oil or a 50:50 mix of hemp and soybean oil at 5%). The effect of dietary oil supplementation to sows was reflected in the plasma fatty acids profile of piglets. Notably the proportion of C20:5n-3 and C22:5n-3 was the highest in plasma of piglets suckling sows fed hemp seed oil-containing diets, whereas no C18:4n-3 could be detected hence indicating conversion of α-linolenic acid (ALA) and stearidonic acid (SDA) to the longer chained n-3 polyunsaturated fatty acids. Dietary fat source also influenced number of born piglets, their weight gain during first week, plasma concentration of glucose and IgG, and haematological profile.

**Conclusions:**

The hemp seed oil resulted in direct maternal supply with n-3 long-chain polyunsaturated fatty acids (LCPUFA), especially ALA and SDA, and piglets were able to convert these fatty acids obtained via the sow milk intake to C20:5n-3 and C22:5n-3. Furthermore, some interesting effects of the 5% hemp seed oil was obtained with regard to piglet initial body weight gain and glucose, which could be of interest for further research, i.e., the capability of hemp seed oil to benefit piglets during early life.

## Background

The sow milk is the main source of nutrients for the piglets during the first weeks of life [1] and plays an essential role in the rate of survival and growth of the piglets. Pigs are born energy deficient and with very low fat deposits [2], but the absorption of lipids and fatty acids from the colostrum and mature milk of the sows is very efficient [3]. Furthermore, maternal antibodies play a crucial role in the protection of neonatal piglets from infectious agents during the first weeks post birth before they develop their own active immunity [4]. It is well recognised that immune components of colostrum and mature milk such as immunoglobulins can be affected by dietary polyunsaturated fatty acids (PUFA) composition [5]. Moreover, it is well established that the fatty acids profile of newborn piglets is affected by the maternal fatty acids intake during pregnancy and lactation [6]. However, when provided the sow during gestation and lactation, more fatty acids reach the piglet through colostrum and mature milk than via the placenta [7]. The dietary essential fatty acids and their long-chain polyunsaturated derivatives are crucial for foetal and postnatal development [8], the function and development of the brain and retina [9], and maintenance of optimal pre- and postnatal growth and development of immunity of newborns [5, 10].

Manipulation of the content and composition of dietary fat in sow diets during late gestation and lactation seems one of the promising methods to influence fat content and composition of colostrum and mature milk, thereby improving the development of the immune system in early life and robustness of their progeny [2, 11]. While a great number of studies described the impact of supplementing different fat sources for sow diets during the late gestation and lactating period with regard to performance of sows and their progeny, there is less evidence in the literature describing the comparison of rapeseed oil, coconut oil, palm oil, sunflower oil, fish oil [2, 12], lard and fish oil [13] or inclusion of various levels of tuna oil [6], olive oil [8, 14, 15] and soybean oil [16] with regard to biological responses of the piglets.

However, in spite of well investigated beneficial impacts of dietary hemp seed oil (hemp oil) supplementation to human health and immunity [17, 18], the literature search revealed very limited amount of studies regarding the application of hemp seed in sow diets [19, 20]. Recent interest focused on hemp seed [21], which is well recognised for its high amount of essential fatty acids, α-linolenic acid (ALA) and linoleic acid (LA) [22]. In addition, intermediate fatty acids such as γ-linolenic acid (GLA) and stearidonic acid (SDA) [17] have been found in hemp seed oil independently from the precursor fatty acids. The presence of these long-chain polyunsaturated fatty acids (LCPUFA) is a unique characteristic for hemp seed because these fatty acids are not obtained in any other common industrial oilseed crop. LCPUFAs play an important role in immune system regulation, blood clotting, neurotransmitter, cholesterol metabolism and structure of membrane phospholipids in the brain and the retina. In addition, hemp seed is well recognised by optimal (for human diets) omega 6 (n-6)/omega 3 (n-3) ratio. This ratio of n-6/n-3 PUFA has a great immunological impact due to its involvement into synthesis of eicosanoids. A previous study by Lauridsen et al. [23] showed that provision of sows with diets differing in n-6/n-3 ratio changed the subsequent relationship in immune cells of the progeny and influenced the eicosanoid synthesis ex vivo*.* A more recent study of Yao et al. [5] indicated that altering the ratio of n-6/n-3 PUFA (from 13:1 to 3:1) in lactating sow diets affected the immune component including immunoglobulin and cytokines of piglets’ plasma and tended to increase the litter average daily gain and improve the immune status of piglets when dietary ratio was 9:1.

In the present research, we investigated the effect of dietary hemp seed oil relative to soybean oil to lactating sows with regard to the transfer of fatty acids to the offspring, and the effect on piglet immunity and their nutritional status. The hypothesis was that dietary provision of hemp seed oil to sows would influence the fatty acid profile of the offspring and their biological responses during suckling.

## Methods

### Experimental design

The experiment was arranged as a randomised complete-block design in which 24 sows [Landrace×Yorkshire] were randomly selected into eight blocks. Within each block, sows had the same parity number (parity = 2, 3, or > 4), and the three experimental dietary treatments were distributed to three sows within each block. The experiment consisted of two blocks of parity 2 and 3, and 5 blocks of parity > 4. Sows had a sire of the same breed, and they were mated with a boar of the Duroc breed. Within each block, sows were mated with the same boar. Cross-fostering of piglets was not performed. Three experimental diets were prepared by mixing of basal diet (standard diet for lactating sows) and oil in ratio 95:5. Each experimental diet included either hemp seed, soybean or a mixture of hemp seed/soybean oil (ratio 50:50). Experimental diets were provided as a mash from day 108 of gestation and during the entire lactating period.

### Animals and housing

At day 108 of gestation, the 24 sows were randomly selected from the herd at the experimental facility at Aarhus University, Foulum, and were moved to the farrowing unit. In the farrowing unit, sows and piglets were housed individually in pens (2.2 m × 2.4 m) with a partly slatted floor, partly solid concrete combined with slats of iron grates, installed heating lamps and with ad libitum access to water. The environmental temperature was initially 21 °C. Sows and piglets were provided with moderate quantities of straw bedding. The animal experiment was conducted according to a license obtained by the Danish Animal Experiments Inspectorate, Ministry of Food, Agriculture and Fisheries, Danish Veterinary and Food Administration, and animals were followed by proper veterinary surveillance throughout the experiment.

### Diets

The experimental diets were provided to the sows from day 108 of gestation and until weaning (4 weeks after farrowing). The sow diet was based on soybean meal, barley and wheat and was composed and mixed at a commercial feed plant (Nutrimin Feed Factory, Ans, Denmark).

The experimental diets were prepared by adding either hemp oil, soybean oil or the mixture of the oils to the basal diet at Research Centre Foulum in ratio 95:5. Hemp seed oil-containing diet (H) was the basal diet with addition of 5% of hemp seed oil; mixed oils-containing diet (HS) was added 5% of a mix of hemp seed and soybean oils (oils in the mix were in ratio 50:50); soybean oil-containing diet (S) was a basal diet with addition of 5% of soybean oil. The provider of the hemp oil was Nørding Olier I/S (Silkeborg, Denmark). The hemp oil contained less than 0.2% ∆9-tetrahydrocannabinol (THC) according to analyses performed at Aalborg University (Esbjerg, Denmark). The soybean oil was a commercial oil available at the pig experimental facility at Aarhus University, Foulum. The basic diet was formulated in order to meet the requirements for sows during late gestation and lactation and to supply an equal amount of nutrients and energy according to Danish Nutrient Standards for Lactating Sows [24]. Before inclusion of the oils to the diets, the fatty acid composition of oils was analysed at Aarhus University, Foulum (Table 1). The contents of dry matter (DM) determined by drying method, crude protein determined by Dumas method, crude fat determined by Nuclear Magnetic Resonance method, and ash of experimental diets were analysed at a commercial company (Eurofins Steins Laboratory A/S, Vejen, Denmark). Dietary lipids were extracted according to Stoldt (1952) [25] using petroleum ether, and the relative composition of fatty acids (> 8 carbons) was determined by gas–liquid chromatography (GLC) after saponification and methylation as described by Rotenberg et al. [26], with substitution of hexane with heptane. Analysis of vitamin E was performed by reverse-phase chromatography (hydrophobic chromatography) as described by Jensen et al. [27]. Sows were fed twice daily and had access to water ad libitum. From day 108 to day 111 of pregnancy, daily rations of 25.5 MJ net energy (NE) were given. Around farrowing, 19.3 MJ NE per day was provided, followed by 23.2 to 27.0 MJ NE on days 2 and 3 post farrowing. Thereafter, feed was offered daily, adjusted to the intake of the individual sow (animals were fed to meet their desired daily intake but could empty the trough between feedings).

**Table 1:** Relative fatty acid composition and vitamin E contents of experimental oils (%)

Fatty acids	Hemp seed oil	Soybean oil
C14:0	0.04	0.08
C15:0	0.02	0.02
C16:0	5.58	10.7
C16:1n-9	0.03	0.01
C16:1n-7	0.10	0.10
C18:0	2.50	2.99
C18:1n-9	10.7	23.0
C18:1n-7	0.82	1.83
C18:2n-6	56.0	53.6
C18:3n-6	3.64	0.03
C18:3n-3	17.4	6.17
C18:4n-3	1.26	0.01
C20:0	0.83	0.31
C20:1n-9	0.39	0.27
C20:2n-6	0.07	0.04
C22:0	0.32	0.44
C24:0	0.13	0.14
Other acids^1^	0.04	0.08
SFA	9.41	14.7
MUFA	11.3	23.4
PUFA	78.5	59.8
Unsaturated fat	89.7	83.2
Total n-6	59.8	53.6
Total n-3	18.7	6.18
Ratio n-6/n-3	3.19	8.68
Vitamin E, mg/kg		
α-tocopherol	38.2	48.1
γ-tocopherol	568	442
δ-tocopherol	22.3	256

^1^Other acids = the sum of C22:1n-9 and C22:1n-11

### Sampling

Blood samples were collected from piglets (four piglets were chosen randomly from each litter) on days 4, 16 and 28 of the lactating period. All blood samples were collected in EDTA-containing and heparinized Vacutainers (Vacuette, Greiner Bio-One GmbH, Kremsmünster, Austria).

After blood sampling, plasma was separated by centrifugation 2,000×*g* and stored at − 20 °C until analysis for fatty acid composition, α-tocopherol, lipids and other metabolites, and determination of immunoglobulins (Ig) G, M, A (IgG, IgM, IgA) concentrations. The colostrum and mature milk samples from each sow were obtained at days 0, 2, 16 and 28 of lactation by hand milking after injection of oxytocin (dose 2 mL per sow). Milk samples were stored at − 20 °C before analysis of fatty acid composition and determination of IgG, IgM, IgA concentrations. One piglet per litter (in total 24 piglets) was euthanized using blunt trauma at day 28 of lactation. After exsanguination, the heart, liver, kidneys and intestine were removed. The intestine was exposed, and clamps were placed in order to separate the proximal, mid and distal part of the small intestine. Further, the small intestine was open within the line of mesenteric attachment, and the content was removed from each part of the small intestine. After emptying the small intestine, samples of the mucus layer and epithelium were obtained by gently scraping at the proximal, mid and distal part of the small intestine for gene expression analysis. Collected tissue and mucus scraping samples were placed in RNA later and frozen at − 80 °C for later analysis.

### Recordings and laboratory analyses

Piglet weight was recorded at birth, and individual sows’ and piglets’ weights were registered with weekly intervals during lactation, i.e., at days 7, 14, 21 and 28. Immediately after collection of blood samples, the analysis of whole blood was performed as a diagnostic health-monitoring tool, using a haematology analyser (IDEXX ProCyte Dx®), and haematological parameters were total leucocytes, neutrophils, lymphocytes, monocytes, eosinophils, erythrocytes, haematocrit, haemoglobin, reticulocytes, thrombocytes, and the mean cell volume, the mean corpuscular haemoglobin and the mean corpuscular haemoglobin concentration. Determination of markers indicating the level of energy and lipid metabolism and nutritional status of the pigs, such as plasma concentrations of glucose, L-lactate, fructosamine and non-esterified fatty acids (NEFA) was performed using an auto analyser (OpeRATMChemistry System, Bayer Corporation, Terrytown, NY, USA), and the procedure was standardised by Technicon RA® Systems as described in Lauridsen et al. [12]. As representative of immune status, the concentration of immunoglobulins A, G and M was measured in plasma of piglets and the colostrum and mature milk of sows using commercial kits (pig ELISA quantitation kit; Bethyl Laboratories, Montgomery TX). The plasma α-tocopherol concentration was analysed as indicator of vitamin E status of pigs, and plasma and sow milk were analysed by high-performance liquid chromatography technique (HPLC) as described by Jensen et al. [27]. Prior to fatty acid analysis of milk, milk samples were thawed and gently mixed in a water bath held at 40 °C. Fat was extracted from the milk according to the method by Bligh and Dyer [28]. To 500 mg of milk was added 0.50 mL of water, 1.00 mL of chloroform with 5,000 mg of C17:0 as internal standard and 2.00 mL of methanol. This monophasic mixture was shaken for 1 min, then 1.00 mL of water and 2.00 mL of chloroform were added, and the mixture was shaken again for 1 min, then 1.00 mL of water and 2.00 mL of chloroform were added, and the mixture was shaken again for 1 min, after which the mixture was centrifuged for 10 min at 1,000×*g* and exactly 1.00 mL of the lower (chloroform) phase was taken out and trans esterified into fatty acid methyl esters after saponification with NaOH and esterified with boron trifluoride methanol complex and separated by GLC as described by Rothenberg and Andersen [26]. The same method was used for determination of plasma fatty acids concentration with the following modifications: 1.00 mL of plasma was used instead of 500 mg of milk, the addition of 0.500 mL of water was excluded, and C17:0 was included as internal standard, and calculation of fatty acid proportions were performed according to this.

The total RNA extraction was performed as described by Sugiharto et al. [29] using the RNAeasy Mini Kit (Qiagen,Hilden,Germany). Quantification of the total RNA was performed spectrophotometrically (NanoDrop Nd-1000 Spectrophotometer, Saveen Werner, Malmö, Sweden). All polymerase chain reactions (PCR) were run in 384-well plates, and the primers listed by Sugiharto et al. [29] were used. The reactions were run in duplicates for the target genes and triplicates for the housekeeping gene using standard amplification conditions determined for the ABI PRISM 7900 real-time PCR system. Gene expression of glyceraldehyde-3-phosphate dehydrogenase (*GAPDH*), cyclooxygenase-2 (*COX-2*), tumour necrosis factor-alpha (*TNF-α*) and interleukin (*IL-10*), as a marker of immunological responses were determined. Gene expression cycle threshold (Ct) values were recorded with the ABI PRISM 7900HT Sequence Detector System (SDS 2.2). *GAPDH* was used as housekeeping gene. The difference in Ct between target gene and *GAPDH* (∆Ct) was used in the statistical analyses.

### Statistical analyses

The statistical analysis was performed according to the following methods by fitting the data to a linear mixed effects model using the *lmer* function from the lme4 package [30] using RStudio (version 1.1.456 for Windows). The impact of diet and production stage on concentrations of fatty acids and of IgG, IgM and IgA in the sows’ colostrum and mature milk, and in piglets’ plasma, piglets’ weight gain, feed intake, haematological parameters and parameters of energy status was investigated by fitting the data to a linear mixed model. Dietary treatments and production stage were included as fixed effects, whereas pig and sow were included as random effects to account for multiple observations made on the same litter and on the same pig. The fixed effects were tested using an F test in which the reduced model was tested against the full model. When a fixed effect was found to be significant, a post hoc test was performed using the multcompView package. Significant interactions between dietary treatments and production stage were obtained with regard to concentration of fatty acids and haematological parameters, and *P*-values are therefore reported for the full model. When a fixed effect was found to be significant, a multiple comparison test was performed using the *lsmeans* package [31]. Effects were considered significant when the probability value *P* was ≤0.05 and considered as ‘trends’ when 0.05 *< P <* 0.10.

## Results

### Dietary content of nutrients

As it can be observed from Table 1, the proportion of total PUFA was greater in hemp seed oil than in soybean oil, and particularly the concentration of α-linolenic acid (ALA, C18:3n-3), γ-linolenic acid (GLA, C18:3n-6), stearidonic acid (SDA, C18:4n-3) and linoleic acid (C18:2n-6) was highest in hemp seed oil. Furthermore, hemp seed oil had the highest proportion of total n-3 fatty acid and was somewhat higher in total n-6 fatty acids than soybean oil, thus the ratio n-6/n-3 was lower in hemp seed oil compared with soybean oil. Soybean oil had a greater proportion of total monounsaturated fatty acids (MUFA) and total saturated fatty acids (SFA). In addition, the concentration of oleic acid (C18:1n-9) and palmitic acid (C16:0) was greater in soybean oil compared to hemp seed oil. The majority of vitamin E in the experimental oils revealed that γ-tocopherol represented the majority and with higher concentration in hemp seed oil than soybean oil, and α-tocopherol and δ-tocopherol were more prevalent in soybean oil. Addition of 5% oils to the experimental diets had provided them with crude fat ranging from 6.35% (hemp seed oil diet) and 6.60% (soybean oil diet) (Table 2). In general, analysis of the fatty acid composition of the experimental diets supplemented with oils reflected the fatty acid composition of the added oil source, i.e., PUFA concentrations increased at the expense of SFA and MUFA when hemp seed oil was added to the diets, and the opposite pattern was observed for diets containing soybean oil. Among the vitamin E-forms, only α-tocopherol could be determined in the experimental diets.

**Table 2:** Ingredients and composition of mean-analysed chemical composition and relative fatty acid composition and vitamin E contents of experimental diets

	Dietary treatment^1^		
	H	HS	S
Ingredients, g/kg of feed			
Wheat, heat treated	508.2	508.2	508.2
Barley, heat treated	237.5	237.5	237.5
Soybean protein concentrate	80.8	80.8	80.8
Vitamin and mineral mix^2^	123.5	123.5	123.5
Oil	50	50	50
Analysed composition			
Dry matter,%	89.9	90.0	90.1
Composition, % of DM			
Crude protein,%	16.0	15.8	15.9
Crude fat,%	6.35	6.55	6.60
Ash,%	5.80	6.05	5.90
Fatty acids, g/100 g DM			
C14:0	0.10	0.11	0.13
C15:0	0.05	0.05	0.05
C16:0	10.8	12.3	14.1
C16:1n-9	0.03	0.02	0.01
C16:1n-7	0.16	0.16	0.16
C18:0	2.46	2.65	2.79
C18:1n-9	12.4	16.3	20.3
C18:1n-7	0.00	0.00	0.03
C18:2n-6	55.1	54.3	53.5
C18:3n-6	2.42	1.33	0.15
C18:3n-3	12.8	9.40	5.67
C18:4n-3	0.79	0.42	0.00
C20:0	0.62	0.46	0.29
Other fatty acids^3^	1.17	1.24	1.16
SFA	14.6	16.1	17.9
MUFA	13.2	17.1	21.1
PUFA	71.2	65.5	59.4
Unsaturated fat	84.4	82.6	80.4
Total n-6	57.6	55.7	53.7
Total n-3	13.6	9.82	5.67
n-6/n-3 ratio	4.25	5.67	9.47
Vitamin E, mg/kg feed			
α-tocopherol	272	340	301

^1^H = basal diet and hemp seed oil in ratio 95:5, HS = basal diet and mix of soybean and hemp seed oils (50:50) in ratio 95:5; S = basal diet and soybean oil in ratio 95:5; basal diet formulated according to Danish Nutrient Standards for Lactating Sows [24]

^2^Supplied per kg of diet (IU/g or mg/kg): 16.6 × 1,000 IU of vitamin A as retinyl acetate; 1.65 × 1,000 IU of vitamin D_3_ as cholecalciferol; 249.2 mg of vitamin E as all-rac-α-tocopherylacetat; 0.7 mg of biotin; 378.9 mg of Fe as FeSO_4_•7H_2_O; 378.9 mg of Fe as C_4_H_2_FeO_4_; 861.4 mg of Cu as CuSO_4_•5H_2_O; 94.4 mg of Mn as MnO; 227.4 mg of Zn as ZnO; 0.9 mg Ca as Ca (IO_3_)_2_; 1.3 mg of Se as Na_2_SeO_3_

^3^Other fatty acids is a sum of C20:1n-9, C20:2n-6, C22:0, C22:1n-11, C22:1n-9, C24:0

### Performance

In general, sows and piglets performed well during the experiment. The experimental diets affected (*P <* 0.001) the number of piglets at birth (Table 3), and the sows on the HS had higher number of total and live born pigs. The number of stillborn pigs ranged from 0.12 (hemp seed oil diet) to 0.49 (soybean oil diet). However, sows’ dietary treatments did not influence number of piglets at weaning. The lactating stage (*P <* 0.001) rather than dietary treatment of sows influenced the sows’ average body weight during the entire experiment, ranging from 320 ± 10.8 kg (hemp seed oil diet) to 346 ± 11.4 kg (soybean oil diet). Piglets’ body weight (Table 3) was influenced by sows’ dietary treatment during the first suckling week (*P =* 0.03); in average, piglets’ weights ranged from 2.66 kg (mix oil diet) to 3.18 kg (hemp seed oil diet). However, sows’ dietary treatments influenced neither birth weight nor piglets’ weights during the subsequent weeks of the suckling period.

**Table 3:** Number of piglets at birth and weaning; sows’ weight changes and weight of piglets during the experimental period

	Dietary treatment^1^			SEM	*P*-value^3^
	H	HS	S		
Number of pigs					
Total born	11.6^b^	14.2^c^	9.63^a^	1.450	< 0.001
Live born	11.6^b^	13.8^c^	9.13^a^	1.420	< 0.001
Stillborn	0.12^a^	0.22^b^	0.49^c^	0.440	< 0.001
At weaning	10.9	11.3	9.51	0.769	0.19
Sows’ initial weight^2^, kg	320	328	333	11.40	0.50
Sows’ weight change, kg	+ 3.87	−1.29	+ 3.08	5.700	0.50
Piglets weight, kg					
At birth	1.75	1.60	1.56	0.100	0.30
Week 1	3.18^a^	2.66^b^	2.88^ab^	0.190	0.03
Week 2	5.25	4.62	4.83	0.310	0.15
Week 3	7.36	6.64	6.70	0.490	0.33
Week 4	9.53	8.42	8.58	0.630	0.22
Weight gain	7.75	6.81	7.07	0.580	0.30

SEM standard error of mean.

^1^H = basal diet and hemp seed oil in ratio 95:5, HS = basal diet and mix of soybean and hemp seed oils (50:50) in ratio 95:5; S = basal diet and soybean oil in ratio 95:5; basal diet formulated according to Danish Nutrient Standards for Lactating Sows [24]

^2^Sows’ weight was registered 24 h after completion of farrowing

^3^In the statistical model, weights were analysed with number of piglets of the sow as covariant

^a,b^Mean values within a row with unlike superscript letters are significantly different

### Fatty acid profile in sows’ milk

Significant interactions between dietary treatments and lactation stage were obtained on concentrations of C18:3n-3, C18:4n-3, C20:4n-6, total MUFAs and PUFAs. The proportion of these fatty acids decreased from day 0 to day 28 of lactation in colostrum and mature milk of sows fed all types of experimental diets, except C18:3n-3 whose concentration increased in colostrum and mature milk of sows fed hemp seed oil diets (Table 4). The highest proportion of C18:3n-3, C18:4n-3 and C20:4n-6 was obtained in milk fat of sows fed hemp seed oil diet when compared with the other two treatments. Dietary oil sources influenced both proportion of MUFAs and PUFAs, i.e., the average percentage of C18:3n-6 and C20:5n-3 was highest in milk fat when hemp seed oil was supplied, whereas the proportion of C16:1n-9 (*P =* 0.02), C18:1n-7 and C22:5n-6 was lowest with soybean oil supplementation. In addition, the concentrations of all fatty acids were significantly influenced by lactation stage. The proportion of total n-3 (*P* < 0.001) and n-6/n-3 ratio (*P <* 0.001) in the colostrum and mature milk was influenced by dietary oil source. The highest proportion of n-3 fatty acids was observed while feeding hemp seed oil diet, and overall the n-6/n-3 ratio was lowest (4.30) in the colostrum and mature milk from sows fed hemp seed oil diet, and the ratio was highest (10.0) when soybean oil was supplemented. It is remarkable that such fatty acids as C10:0, C12:0, C20:3n-6, C20:4n-6, C20:5n-3, C22:5n-6, C22:5n-3 and C22:6n-3 were detected in sows’ milk fat but were under quantification level in the feed.

**Table 4:** Relative proportion of selected fatty acids in milk (%)

	Dietary treatment^1^												SEM	*P-*value	
	H				HS				S					Diet	Day^2^ × Diet
Day of lactation	0	2	16	28	0	2	16	28	0	2	16	28			
SFA	26.2	27.6	38.8	38.6	25.7	27.0	38.2	38.1	25.9	27.3	38.4	38.3	1.093	0.85	0.67
C14:0	1.27	1.53	3.53	3.56	1.12	1.38	3.37	3.40	1.05	1.32	3.31	3.35	0.155	0.26	0.69
C16:0	19.5	20.4	30.8	31.24	30.6	18.8	19.6	30.0	20.2	21.1	31.5	31.3	0.896	0.07	0.08
C18:0	4.93	5.30	4.12	3.93	4.87	5.25	4.06	3.87	4.75	5.13	3.94	3.75	0.245	0.68	0.73
MUFA	37.8^bc^	41.4^c^	30.3^ab^	26.3^a^	36.4^abc^	40.3^bc^	35.4^abc^	36.4^abc^	37.0^abc^	38.7^abc^	32.4^abc^	29.3^abc^	3.283	0.02	0.05
C16:1n-9	1.20^a^	1.05^a^	0.32^a^	0.26^a^	1.33^b^	1.18^b^	0.44^b^	0.39^b^	1.24^ab^	1.08^ab^	0.35^ab^	0.29^ab^	0.054	0.02	0.32
C16:1n-7	2.33	3.28	8.23	7.66	2.37	3.32	8.27	7.70	2.13	3.07	8.03	7.45	0.569	0.88	0.36
C18:1n-9	30.2	33.0	23.1	22.2	31.9	34.7	24.8	23.9	28.2	31.0	21.1	20.2	2.442	0.26	0.12
C18:1n-7	2.67^b^	2.78^b^	1.87^a^	1.70^a^	2.96^b^	3.06^b^	2.16^a^	1.99^a^	2.75^b^	2.85^b^	1.94^a^	1.77^a^	0.153	0.04	0.10
PUFA	35.2^ab^	29.6^ab^	28.9^ab^	34.1^ab^	36.1^b^	30.9^ab^	25.6^ab^	23.9^a^	36.5^ab^	34.3^ab^	26.6^ab^	30.5^ab^	3.371	0.03	0.05
C18:2n-6	28.9	24.8	20.5	22.3	28.9	24.7	20.4	22.2	32.5	28.4	24.7	25.9	2.429	0.26	0.15
C18:3n-6	1.16^b^	0.94^b^	0.66^b^	0.77^b^	0.87^a^	0.65^a^	0.37^a^	0.48^a^	0.65^a^	0.44^a^	0.16^a^	0.26^a^	0.110	0.002	0.14
C18:3n-3	4.43^b^	3.48^a^	3.76^b^	5.31^b^	3.41^a^	2.81^a^	2.74^a^	2.52^a^	2.63^a^	2.40^a^	2.29^a^	2.32^a^	0.667	0.003	0.03
C18:4n-3	0.24^c^	0.20^bc^	0.14^ab^	0.19^abc^	0.13^abc^	0.11^abc^	0.08^abc^	0.06^ab^	0.01^ab^	0.03^ab^	0.01^ab^	0.00^a^	0.037	< 0.001	0.04
C20:2n-6	0.43	0.45	0.36	0.33	0.44	0.46	0.37	0.34	0.48	0.50	0.41	0.38	0.029	0.15	0.10
C20:3n-6	0.81	0.73	0.56	0.43	0.86	0.78	0.61	0.47	0.95	0.87	0.70	0.57	0.077	0.14	0.80
C20:4n-6	0.18^a^	0.16^b^	0.17^b^	0.17^b^	0.16^b^	0.16^b^	0.12^b^	0.12^b^	0.14^b^	0.13^b^	0.09^a^	0.10	0.021	0.003	0.01
C20:5n-3	0.15^c^	0.13^c^	0.11^a^	0.11^a^	0.14^c^	0.11^b^	0.09^a^	0.09^a^	0.10^b^	0.08^a^	0.06^a^	0.06^a^	0.016	0.03	0.95
C22:5n-6	0.10^a^	0.09^a^	0.10^a^	0.05^a^	0.12^a^	0.11^a^	0.12^a^	0.07^a^	0.17^b^	0.16^b^	0.17^b^	0.12^b^	0.019	0.001	0.34
C22:5n-3	0.41	0.35	0.27	0.21	0.46	0.40	0.31	0.25	0.46	0.39	0.31	0.25	0.045	0.43	0.96
C22:6n-3	0.06	0.05	0.02	0.02	0.07	0.06	0.03	0.03	0.05	0.05	0.02	0.01	0.011	0.20	0.42
n-6	32.1	28.1	23.8	25.8	29.4	25.5	21.2	23.2	33.1	29.1	24.8	26.8	1.925	0.13	0.06
n-3	5.94^a^	5.27^a^	5.32^a^	5.85^a^	4.15^b^	3.48^b^	3.53^b^	4.06^b^	3.47^c^	2.80^c^	2.86^c^	3.38^c^	0.471	< 0.001	0.07
n-6/n-3	5.59^a^	5.75^a^	4.62^a^	4.30^a^	7.25^b^	7.41^b^	6.28^b^	5.97^b^	9.89^c^	10.0^c^	8.92^c^	8.60^c^	0.496	< 0.001	0.96

SEM standard error of mean

^1^H = basal diet and hemp seed oil in ratio 95:5, HS = basal diet and mix of soybean and hemp seed oils (50:50) in ratio 95:5; S = basal diet and soybean oil in ratio 95:5; basal diet formulated according to Danish Nutrient Standards for Lactating Sows [24]

^2^Effect of day is significant for all lactation stages, except total n-3 fatty acids *P* = 0.12

^a,b,c^Mean values within a row with unlike superscript letters are significantly different

**Table 5:** Composition of selected^2^ fatty acids of sows’ plasma (%).

	Dietary treatment^1^															SEM	*P*-value	
	H					HS					S						Diet	Day^4^ × Diet
Day^2^	108	112	2	16	28	108	112	2	16	28	108	112	2	16	28			
SFA	30.4	28.2	29.1	29.4	29.4	29.7	29.5	28.5	28.7	28.7	29.8	27.6	28.5	28.8	28.8	0.403	0.18	0.06
C14:0	0.42	0.30	0.28	0.23	0.25	0.43	0.31	0.29	0.24	0.26	0.45	0.33	0.31	0.26	0.27	0.020	0.31	0.45
C16:0	17.0	13.7	13.6	14.7	14.6	17.0	13.8	13.7	14.8	14.7	17.6	14.4	14.3	15.4	15.3	0.329	0.07	0.01
C18:0	12.0^b^	13.1^b^	14.0^b^	13.4^b^	13.7^b^	11.3^ab^	12.5^ab^	13.4^ab^	12.8^ab^	13.1^ab^	10.7^a^	11.9^a^	12.8^a^	12.3^a^	12.5^a^	0.324	0.004	0.90
MUFA	28.2	20.2	22.7	15.4	15.0	28.5	21.3	22.5	17.9	19.8	27.3	22.7	26.6	20.0	21.8	1.510	0.003	0.009
C16:1n-9	0.48^b^	0.41^b^	0.43^b^	0.26^a^	0.29^a^	0.56^b^	0.49^b^	0.50^b^	0.33^a^	0.36^a^	0.55^b^	0.48^b^	0.50^b^	0.33^a^	0.36^a^	0.034	0.03	0.30
C16:1n-7	1.10	0.82	0.96	0.67	0.70	1.16	0.88	1.02	0.77	1.01	1.12	0.83	0.98	0.68	0.72	0.083	0.31	0.10
C18:1n-9	23.5^a^	16.4^a^	18.3^a^	12.4^a^	12.3^a^	23.6^ab^	17.4^ab^	18.1^ab^	14.6^ab^	16.3^ab^	22.8^b^	18.7^b^	21.8^b^	16.4^b^	18.4^b^	1.276	0.03	0.005
C18:1n-7	2.25	1.92	2.26	1.60	1.49	2.37	2.02	2.37	1.72	1.61	2.39	2.04	2.40	1.74	1.63	0.137	0.59	0.42
PUFA	40.8	51.6	47.9	55.1	56.3	42.0	51.3	49.0	53.3	51.1	43.5	49.6	45.0	51.5	48.7	1.600	0.001	0.002
C18:2n-6	28.8	35.6	30.5	40.6	41.8	28.8	36.1	32.1	39.8	37.8	30.4	36.1	30.9	39.7	38.0	1.572	0.90	0.25
C18:3n-6	1.34	1.76	2.07	1.55	1.48	2.84	2.63	1.68	2.08	2.01	1.63	1.63	2.51	1.49	1.13	0.470	0.76	0.82
C18:3n-3	1.30	2.76	2.09	3.14	3.22	1.14	2.10	1.55	2.06	1.77	1.42	1.64	1.22	1.43	1.26	0.207	< 0.001	< 0.001
C18:4n-3	0.08^b^	0.10^b^	0.11^b^	0.10^b^	0.11^b^	0.04^a^	0.07^b^	0.07^b^	0.07^b^	0.08^b^	0.00^a^	0.01^a^	0.02^a^	0.01^a^	0.03^a^	0.019	0.001	0.16
C20:2n-6	0.20	0.25	0.24	0.27	0.29	0.20	0.22	0.23	0.18	0.20	0.18	0.23	0.22	0.25	0.27	0.018	0.14	0.77
C20:3n-6	0.46	0.44	0.45	0.58	0.68	0.39	0.37	0.37	0.51	0.61	0.30	0.27	0.28	0.41	0.51	0.085	0.17	< 0.001
C20:4n-6	6.75	7.39	8.33	5.97	5.85	7.11	7.75	8.69	6.33	6.21	6.59	7.23	8.17	5.81	5.69	0.424	0.50	0.40
C20:3n-3	0.03	0.10	0.02	0.09	0.14	0.01	0.04	0.00	0.05	0.05	0.01	0.01	0.04	0.03	0.01	0.016	< 0.001	< 0.001
C20:5n-3	0.39	1.02	1.08	1.20	1.16	0.34	0.65	0.75	0.81	0.94	0.38	0.43	0.43	0.45	0.53	0.088	< 0.001	< 0.001
C22:5n-6	0.29	0.23	0.09	0.09	0.07	0.31	0.20	0.27	0.14	0.12	0.27	0.26	0.28	0.21	0.19	0.042	0.002	0.01
C22:5n-3	1.40	1.65	2.01	1.48	1.35	1.46	1.60	1.86	1.61	1.49	1.37	1.55	1.41	1.30	1.21	0.112	0.003	0.01
C22:6n-3	0.34	0.32	0.36	0.23	0.18	0.34	0.32	0.36	0.23	0.18	0.30	0.28	0.31	0.19	0.13	0.052	0.62	0.08
n-6	37.3	45.8	42.2	49.0	50.3	38.7	46.6	44.5	48.5	46.5	40.0	45.7	41.7	48.1	45.6	1.463	0.07	0.03
n-3	3.46	5.83	5.68	6.18	6.07	3.56	4.00	4.53	4.79	4.54	3.56	3.92	3.35	3.42	3.11	0.250	< 0.001	< 0.001
n-6/n-3	11.1	7.94	7.49	7.97	8.36	11.8	9.78	9.88	10.2	10.3	11.3	11.6	12.8	14.2	14.6	0.518	< 0.001	< 0.001

SEM standard error of mean.

^1^H = basal diet and hemp seed oil in ratio 95:5, HS = basal diet and mix of soybean and hemp seed oils (50:50) in ratio 95:5; S = basal diet and soybean oil in ratio 95:5; basal diet formulated according to Danish Nutrient Standards for Lactating Sows [24]

^2^Day implies the sampling at respective days of the gestation period (108 and 112), and days of lactation period (2, 16, 28)

^3^Such fatty acids as C4:0, C8:0, C10:0, C12:0,C15:0, C17:1, C20:0,C22:0 were present (< 0.3%)

^4^Effect of day is significant for all sampling days, except C18:4n-3 *P* > 0.05

^a,b^Mean values within a row with unlike superscript letters are significantly different

### Fatty acid profile in plasma of sows

The physiological stage and dietary treatment of sows influenced the fatty acid composition in sow plasma, and interactions between the treatments were observed on several of the fatty acids in sows’ plasma (Table 5). Arachidonic acid (ARA) was influenced by day of sampling, whereas dietary treatments of sows had no influence on the concentration of this fatty acid. Additionally, the total n-3 and n-6 fatty acid concentration was observed in the highest proportion while feeding hemp seed oil diet. The n-6/n-3 ratio in sow plasma was influenced by significant interactions between day of sampling and dietary treatment. The n-6/n-3 ratio was lowest (7.49) when fed hemp seed oil diet, and a decrease was observed with progressing lactation stage when sows received the hemp seed oil diet, whereas the opposite pattern was observed regarding n-6/n-3 ratio in sow plasma when fed soybean oil diet. Dietary treatment of sows had a significant effect on average concentrations of C18:0 (*P* = 0.004) being highest in sows fed hemp seed oil diet (14.0% of total fat) in comparison with sows on soybean oil diet (10.7% of total fat). The C16:1 concentration was highest (*P =* 0.03) in sows’ plasma when fed soybean oil-including diets, and the C18:4n-3 concentration (*P =* 0.001) was highest in sows fed hemp oil-including diets. Short-chained fatty acids as C4:0 and C8:0 were detected in sows’ plasma despite the fact that they were not detectable in diets and sow milk.

### Fatty acid profile in plasma of piglets

Significant interactions between dietary treatment of sows and age of piglets were observed on C16:0 (*P <* 0.001), C18:3n-6 (*P =* 0.03), C18:3n-3 (*P <* 0.001) and total SFA (*P <* 0.001), i.e., the proportion of C16:0 and C18:3n-3 increased from day 4 to day 28 of age, and the highest proportion of these acids was observed in plasma of piglets from sows allotted either hemp seed oil or soybean oil diets (Table 6). With regard to the proportion of C18:3n-6 and total SFA, the proportion of these acids decreased in plasma of piglets from sows fed mixed oil diet. The source of dietary oil fed to the sows influenced markedly the total MUFA (*P =* 0.04) and the total PUFA (*P =* 0.03) in plasma of piglets, i.e., the highest proportion of MUFA was observed in piglets’ plasma when fed soybean oil-containing diets, whereas the highest proportion of PUFA was observed in piglets’ plasma when fed hemp seed oil-containing diets. Furthermore, the effect of dietary oil supplementation of sows was reflected in piglets’ plasma fatty acids profile, i.e., the proportion of the following acids C18:0 (*P =* 0.03), C20:3n-6 (*P <* 0.001), C20:5n-3 (*P <* 0.001) and C22:5n-3 (*P <* 0.001) was highest in plasma of piglets suckling sows fed hemp seed oil-containing diets, whereas the soybean oil contributed to an elevated proportion of C18:1n-9 (*P =* 0.02) in piglets’ plasma. Hemp seed oil supplementation increased (*P <* 0.001) the proportion of total n-3 compared with the other oil supplements. The n-6/n-3 ratio was influenced by dietary treatment of sows (*P <* 0.001) and ranged in average from 7.42 (hemp oil diet) to 17.7 (soybean oil diet).

**Table 6:** Composition of selected^2^ fatty acids of piglets’ plasma (%)

	Dietary treatment^1^									SEM	*P*-value	
	H			HS			S				Diet	Day^3^×Diet
Day of age	4	16	28	4	16	28	4	16	28			
SFA	34.6	39.2	36.4	36.3	36.6	35.3	35.1	37.8	35.8	0.498	< 0.001	< 0.001
C14:0	0.65	0.73	0.84	0.80	0.88	0.99	0.81	0.88	0.99	0.098	0.31	0.58
C16:0	20.0	25.3	24.3	21.8	23.3	23.9	21.6	25.4	24.2	0.754	0.002	< 0.001
C18:0	13.2^d^	12.4	10.8^b^	12.4^c^	11.7^c^	10.0^a^	11.8^c^	11.1^b^	9.47^a^	0.378	0.03	0.78
MUFA	24.3^c^	17.0^a^	15.3^a^	27.2^c^	19.8^b^	18.2^b^	29.5^c^	22.1^b^	20.5^b^	1.504	0.04	0.58
C16:1n-7	2.34	3.40	3.23	2.65	3.71	3.54	2.36	3.42	3.26	0.247	0.42	0.08
C16:1n-9	0.57	0.25	0.29	0.60	0.28	0.33	0.61	0.29	0.33	0.055	0.79	0.53
C18:1n-9	18.6bcd	11.2^a^	10.1^a^	20.7 ^cd^	13.3^ab^	12.3^ab^	23.2^d^	15.9^abc^	14.8^abc^	1.224	0.02	0.29
C18:1n-7	2.45	1.74	1.44	2.67	1.95	1.65	2.74	2.02	1.72	0.127	0.16	0.93
PUFA	39.4^a^	44.2^b^	47.8^c^	37.5^a^	42.3^b^	45.9^c^	34.8^a^	39.6^b^	43.2^c^	1.300	0.03	0.82
C18:2n-6	29.2	33.7	34.4	27.4	31.9	32.7	26.6	31.1	31.8	1.603	0.38	0.54
C18:3n-6	0.42	0.52	0.72	0.70	1.63	0.26	1.15	0.36	2.34	0.923	0.07	0.03
C18:3n-3	1.76	2.01	2.71	1.42	1.81	1.90	0.90	0.99	1.14	0.148	< 0.001	< 0.001
C20:3n-6	0.57^c^	0.56^c^	0.55^c^	0.47^b^	0.47^b^	0.45^b^	0.28^a^	0.27^a^	0.26^a^	0.035	< 0.001	0.26
C20:4n-6	5.03	4.88	6.15	5.15	5.00	6.28	4.58	4.43	5.71	0.366	0.35	0.92
C20:5n-3	0.28^c^	0.28^c^	0.45^c^	0.16^b^	0.16^b^	0.34^c^	0.00^a^	0.004^a^	0.17^b^	0.031	< 0.001	0.26
C22:1n-9	0.40	0.38	0.25	0.53	0.50	0.37	0.52	0.49	0.37	0.149	0.70	0.12
C22:5n-3	0.84^b^	0.92^b^	1.26^b^	0.78^b^	0.86^b^	1.20^b^	0.49^a^	0.56^a^	0.91^a^	0.062	< 0.001	0.79
C22:6n-3	0.80	0.93	1.32	0.87	1.00	1.40	0.57	0.70	1.10	0.116	0.08	0.82
n-6	35.6	39.9	42.3	34.2	38.6	41.0	33.0	37.4	39.8	1.246	0.23	0.69
n-3	3.84 ^cd^	4.27 ^cd^	5.46^e^	3.30^bc^	3.73^c^	4.93^de^	1.83^a^	2.26^ab^	3.45^c^	0.204	< 0.001	0.15
n-6/n-3	9.64^ab^	9.30^ab^	7.42^a^	10.6^abc^	10.2^ab^	8.34^a^	17.7^d^	17.4 ^cd^	15.5^bcd^	1.202	< 0.001	0.10

SEM standard error of mean

^1^H = basal diet and hemp seed oil in ratio 95:5, HS = basal diet and mix of soybean and hemp seed oils (50:50) in ratio 95:5; S = basal diet and soybean oil in ratio 95:5; basal diet formulated according to Danish Nutrient Standards for Lactating Sows [24]

^2^Such fatty acids as C4:0, C8:0, C10:0, C12:0, C15:0, C16:1n-9, C17:1, C20:0, C22:0 were also present (concentration < 0.3%)

^3^Effect of day is significant for all sampling days, except C18:3n-6, C20:3n-6, C22:1n-9 and n-6/n-3 ratio *P* > 0.05

^a,b,c,d,e^Mean values within a row with unlike superscript letters are significantly different

### Concentration of α-tocopherol in plasma of sows and piglets

Day of sampling influenced α-tocopherol concentrations in sows’ plasma during late gestation and lactation, and piglet’s age influenced the α-tocopherol concentration in plasma of piglets during the suckling period (Table 7). The concentration of α-tocopherol decreased from day 108 of gestation to day 2 of lactation in plasma of sows in all dietary treatments and subsequently increased from day 2 to day 28 of lactation. The concentration of α-tocopherol in piglets’ plasma decreased as age of piglets increased (*P <* 0.001). A tendency to a lower concentration of plasma α–tocopherol was observed in plasma of piglets suckling sows on the mixed oil dietary treatment compared with the other treatments.

**Table 7:** The concentration of α-tocopherol, mg/L, in plasma of sows and piglets

	Dietary treatment^1^			SEM
	H	HS	S	
Day^2^ of gestation/lactation of sows				
108	2.35^ab^	2.70^ab^	2.70^ab^	0.201
112	2.17^a^	2.51^a^	2.52^a^	0.208
2	1.82^a^	2.17^a^	2.17^a^	0.201
16	2.85^bc^	3.20^bc^	3.20^bc^	0.201
28	3.33^c^	3.68^c^	3.68^c^	0.203
Day^3^ of age of piglets				
4	5.22^c^	4.50^c^	5.54^c^	0.412
16	4.35^b^	3.63^b^	4.66^b^	0.412
28	2.52^a^	1.80^a^	2.83^a^	0.412

SEM standard error of mean.

^1^H = basal diet and hemp seed oil in ratio 95:5, HS = basal diet and mix of soybean and hemp seed oils (50:50) in ratio 95:5; S = basal diet and soybean oil in ratio 95:5; basal diet formulated according to Danish Nutrient Standards for Lactating Sows [24]

^2^Effect of day *P <* 0.001 and effect of dietary treatment *P* = 0.13. No significant interaction was observed

^3^Effect of day *P <* 0.001 and effect of dietary treatment *P* = 0.09. No significant interaction was observed

^a,b,c^Mean values within a column of sows or piglets response with unlike superscript letters are significantly different

### Concentration of immunoglobulins in sows’ milk

**Fig. 1:**
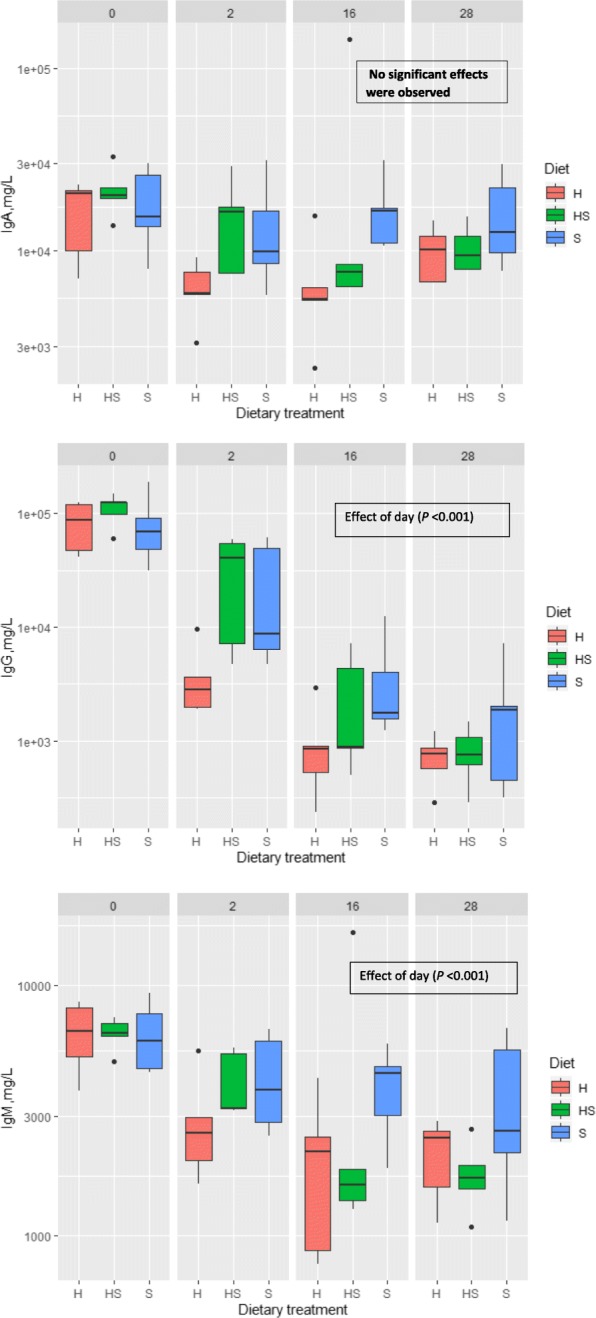
Concentration of immunoglobulins IgM, IgG and IgA, mg/L in colostrum and mature milk of sows (days 0, 2, 16 and 28 of lactation). H = basal diet and hemp seed oil in ratio 95:5, HS = basal diet and mix of soybean and hemp seed oils (50:50) in ratio 95:5; S = basal diet and soybean oil in ratio 95:5; basal diet formulated according to Danish Nutrient Standards for Lactating Sows [24]

The concentration of IgG and IgM in sows’ milk was influenced by lactation stage (*P <* 0.001) (Fig. 1), as concentration of these immunoglobulins decreased with progressed lactation. The dietary treatments allotted to sows influenced significantly the IgG concentration *(P =* 0.02) and tended to influence the IgA concentration (*P =* 0.07) in sows’ milk at day 2 of lactation and IgM (*P =* 0.09) concentration in sows’ mature milk at day 28 of lactation. With regard to the IgG concentration, the observed average concentration ranged from 5714 mg/L (hemp seed oil diet) to 12,066 mg/L (soybean oil diet) and 14,353 mg/L (mixed oil diet) in sows’ milk at day 2 of lactation. In general, the average concentration of IgG, IgA and IgM was lower during the whole lactation period in sows’ colostrum and mature milk when fed the hemp seed oil diet compared to two other experimental diets.

### Concentration of immunoglobulins in plasma of piglets

**Fig. 2:**
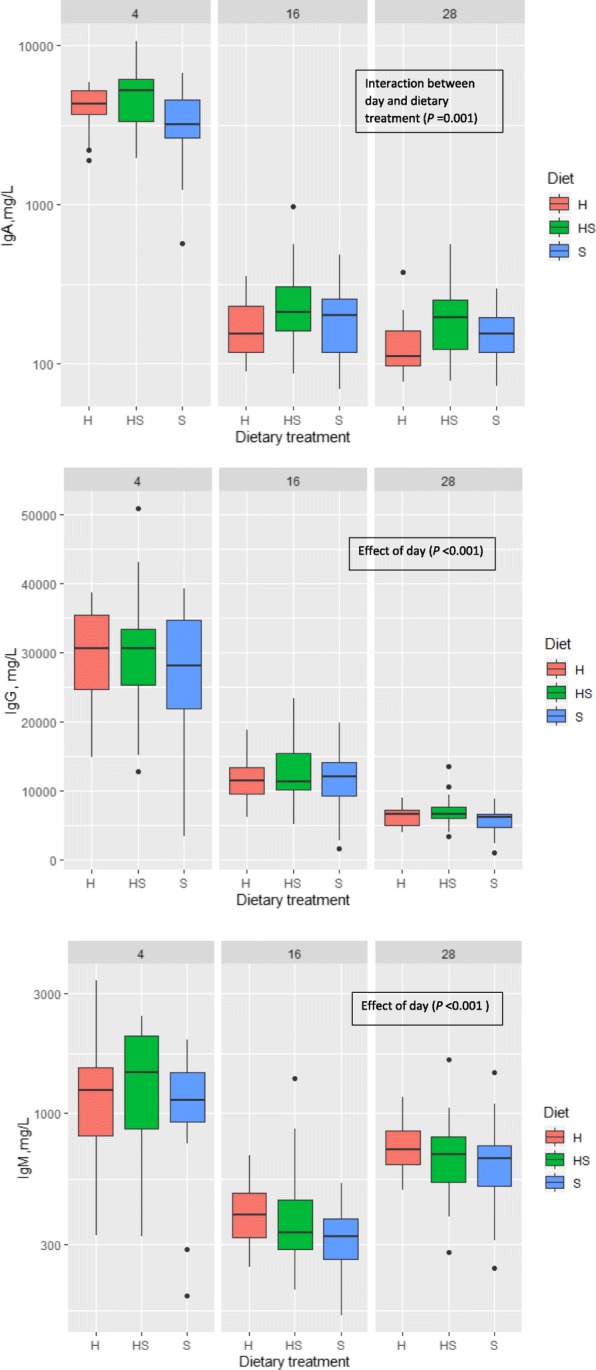
Concentration of immunoglobulins IgM, IgG and IgA, mg/L in piglets’ plasma at birth and on days 2, 16 and 28 of age. H = basal diet and hemp seed oil in ratio 95:5, HS = basal diet and mix of soybean and hemp seed oils (50:50) in ratio 95:5; S = basal diet and soybean oil in ratio 95:5;basal diet formulated according to Danish Nutrient Standards for Lactating Sows [24]

The analysis of immunoglobulin composition of piglets’ plasma revealed that the concentration of IgG, IgM and IgA in piglets’ plasma was influenced by piglets’ age (*P <* 0.001) (Fig. 2), i.e., higher concentration of mentioned immunoglobulins in piglets’ plasma at day 4 of age compared with subsequent days of age. A significant interaction (*P =* 0.001) between dietary treatment and age of piglets was obtained on IgA in piglets’ plasma, thus concentration of IgA was higher in piglets suckling sows on hemp oil than sows provided soybean oil at day 4 of age, but lower during later age. Concentrations of IgM (*P =* 0.03) and IgG (*P =* 0.008) were influenced by the dietary oil source fed to sows at days 16 and 28 of age, respectively. Thus, piglets from sows fed mixed oil diet had, in average, the highest concentration of IgM (458 mg/L), and the piglets from sows fed soybean oil diet the lowest (312 mg/L). The same pattern was observed for IgG concentration in plasma of piglets of sows fed the mixed oil diet (7547 mg/L) and soybean oil diet (5463 mg/L).

### Nutritional status of piglets

The nutritional status of piglets assessed by metabolites, such as beta-hydroxybutyrate (BHB), non-esterified fatty acids (NEFA), phospholipids, glucose, triglycerides, L-lactate, cholesterol and fructosamine in piglets’ plasma during the suckling period is shown in Table 8. Among these metabolites, only the concentration of glucose was influenced by the dietary treatment (*P =* 0.01) allotted to sows, i.e., the highest average concentration of glucose (7.21 mmol/L) during the entire experiment was observed in the plasma of piglets suckling sows fed the hemp seed oil diet and the lowest (6.64 mmol/L) in piglets of sows fed the mixed oil diet. Age of piglets influenced the concentrations of glucose, NEFA, phospholipids, cholesterol and fructosamine (*P* < 0.001), as the concentrations of NEFA and fructosamine increased in piglets’ plasma, whereas phospholipids and cholesterol concentrations decreased in piglets’ plasma with progressing of age.

**Table 8:** Blood plasma concentrations of beta-hydroxybutyrate (BHB), non-esterified fatty acids (NEFA), phospholipid, glucose,triglyceride, *L*-lactate, cholesterol and fructosamine in piglets

	Dietary treatment^1^									SEM	*P*-value^3^
	H			HS			S				
Day^2^ of age	4	16	28	4	16	28	4	16	28		
BHB, mmol/L	0.08	0.07	0.09	0.08	0.07	0.08	0.09	0.08	0.10	0.008	0.37
NEFA, μEq/L	279	332	436	305	357	462	332	385	489	27.40	0.20
Phospholipid, mmol/L	2.12	2.34	1.87	2.19	2.40	1.93	2.29	2.51	2.03	0.081	0.16
Glucose^4^, mmol/L	6.58^a^	7.70^b^	7.36^a^	6.01^b^	7.12^b^	6.79^b^	6.28^a^	7.40^b^	7.06^b^	0.179	0.01
Triglyceride, mmol/L	0.92	0.71	0.87	0.87	0.66	0.83	0.94	0.74	0.90	0.126	0.85
*L*-lactate, mmol/L	6.12	6.53	7.16	4.79	5.20	5.82	6.15	6.56	7.18	0.645	0.12
Cholesterol, mmol/L	3.53	4.54	3.04	3.58	4.58	3.08	3.68	4.69	3.19	0.236	0.84
Fructosamine, μmol/L	212	228	242	197	214	228	207	224	239	6.609	0.20

SEM standard error of mean

^1^H = basal diet and hemp seed oil in ratio 95:5, HS = basal diet and mix of soybean and hemp seed oils (50:50) in ratio 95:5; S = basal diet and soybean oil in ratio 95:5; basal diet formulated according to Danish Nutrient Standards for Lactating Sows [24]

^2^Effect of day observed significant *P* < 0.001, for NEFA, phospholipid, glucose, cholesterol and fructosamine; however, for BHB, triglyceride and L-lactate was not observed the significant effect of day *P* > 0.05

^3^Presented *P*-value attributed to effect of dietary treatment

^4^The average concentration of glucose in piglets’ plasma during the entire experiment: 7.21 mmol/L (a), 6.64 mmol/L (b) and 6.91 mmol/L (ab) suckled sows fed hemp seed oil, mix oil and soybean oil, respectively

^a,b^Mean values within a row with unlike superscript letters are significantly different

### Haematological parameters of piglets

Significant interactions between dietary treatment of sows and age of piglets were observed on the proportion of eosinophils (*P =* 0.001) in total white blood cells, erythrocytes concentration (*P =* 0.03) and mean cell volume (*P =* 0.02) in piglets’ blood (Table 9). Eosinophils and erythrocytes increased with piglets’ age, and the highest values for these haematological parameters were observed in piglets from sows fed the mixed oil diet. The monocytes proportion in piglets’ total white blood cells was influenced by dietary treatments of sows (*P =* 0.04), i.e., the highest percentage (4.75%) of monocytes in total white blood cells was observed in piglets’ blood in pigs suckling sows provided with soybean oil diet, and the lowest (4.04%) was observed in piglets from sows fed the mixed oil diet. In addition, all haematological parameters, such as concentrations of white blood cells (WBC), neutrophils, lymphocytes, monocytes, eosinophils, erythrocytes, reticulocytes, thrombocytes, haemoglobin, neutrophils to lymphocytes ratio (NLR), haematocrit, the mean cell volume (MCV), the mean corpuscular haemoglobin (MCH) and the mean corpuscular haemoglobin concentration (MCHC) were influenced by age of piglets (*P <* 0.001).

**Table 9:** Haematological parameters of piglets

	Dietary treatment^1^									*P*-value	
	H			HS			S			Diet	Day^2^ × Diet
Day of age	4	16	28	4	16	28	4	16	28		
WBC, ×10^9^/L	12.6^c^	8.27^a^	10.1^b^	12.5^c^	8.14^a^	9.95^b^	13.1^c^	8.70^a^	10.5^b^	0.60	0.39
Neutrophils, %	51.7^b^	34.6^a^	36.1^a^	64.1^b^	37.0^a^	38.5^a^	54.3^b^	37.2^a^	38.7^a^	0.13	0.37
Lymphocytes, %	42.4^a^	61.0^c^	58.2^b^	40.6^a^	59.3^c^	56.5^b^	39.7^a^	58.3^c^	55.5^b^	0.22	0.53
NLR	0.18^b^	0.05^a^	0.07^a^	0.19^b^	0.07^a^	0.08^a^	0.20^b^	0.07^a^	0.09^a^	0.42	0.32
Monocytes, %	5.28^de^	3.62^ab^	4.83^cde^	4.75^cde^	3.09^a^	4.30^bcd^	5.45^e^	3.79^abc^	5.00^de^	0.04	0.38
Eosinophils, %	0.45^a^	0.60^abc^	0.67^abc^	0.45^a^	0.81^bc^	0.84^c^	0.62^abc^	0.55^ab^	0.68^abc^	0.002	0.001
Erythrocytes, ×10^12^/L	3.91^a^	5.57^bc^	6.19^d^	3.83^a^	5.60^bc^	6.40^d^	3.91^a^	5.20^b^	6.05^cd^	0.01	0.03
Haematocrit, %	24.8^a^	36.2^c^	33.3^b^	25.0^a^	36.3^c^	33.5^b^	23.9^a^	35.2^c^	32.3^b^	0.41	0.18
Haemoglobin, mmol/L	70.8^a^	90.1^cfg^	93.9^bde^	72.6^a^	101^eg^	95.7^bcdf^	68.7^a^	97.0^defg^	91.8^bc^	0.28	0.06
MCV, ×10^−15^ L	64.1^bd^	67.7^ce^	53.5^a^	64.0^bcde^	64.9^bcde^	53.1^a^	63.7^bc^	67.5^de^	52.5^a^	0.06	0.02
MCH, pg/mL	18.4^b^	18.3^b^	15.1^a^	18.4^b^	18.3^b^	15.1^a^	18.4^b^	18.3^b^	15.1^a^	0.10	0.13
MCHC, g/L	286^b^	274^a^	285^b^	289^b^	277^a^	287^b^	287^b^	274^a^	284^b^	0.28	0.19
Reticulocytes, %	6.78^c^	5.89^b^	1.59^a^	7.38^c^	6.48^b^	2.18^a^	6.76^c^	5.85^b^	1.56^a^	0.12	0.22
Thrombocytes, ×10^9^/L	466^a^	542^b^	606^c^	492^a^	568^b^	633^c^	475^a^	552^b^	616^c^	0.44	0.52

WBC total white blood cells, NLR – neutrophils to lymphocytes ratio, MCV the mean cell volume-the ratio of the haematocrit to the concentration of red blood cells, MCH the mean corpuscular haemoglobin-the ratio of the total mass of haemoglobin to the number of red blood cells, MCHC the mean corpuscular haemoglobin concentration - identifies the amount of haemoglobin in a single red blood cell

^1^H = basal diet and hemp seed oil in ratio 95:5, HS = basal diet and mix of soybean and hemp seed oils (50:50) in ratio 95:5; S = basal diet and soybean oil in ratio 95:5; basal diet formulated according to Danish Nutrient Standards for Lactating Sows [24]

^2^Effect of day is significant for all ages

^a,b,c,d,e,f,g^Mean values within a row with unlike superscript letters are significantly different

### Intestinal gene expression of COX-2, IL-10, TNF-α

In general, gene expression of *COX-2, IL-10* and *TNF-α* in piglets’ intestine differed significantly between site of intestinal segments (proximal, mid distal small intestine) and type of tissue (mucus layer and intestinal epithelium) (Appendix 1). Thus, the highest cycle threshold (Ct) of *COX-2, IL-10* and *TNF-α* was found in proximal and mid part of the intestine, whereas the lowest Ct value was detected in the distal part of the small intestine of all piglets from sows fed experimental diets (*P <* 0.001). With regard to type of tissue, the analysis revealed the highest Ct value in the intestinal mucus independently from experimental diet type, and the lowest Ct value was detected in the intestinal epithelium (*P <* 0.001) for all experimental diets. In addition, the *IL-10* gene expression in intestinal epithelium tended to be influenced by dietary treatments (*P =* 0.09), thus the highest Ct values were observed in all segments of the small intestine of piglets from sows fed soybean oil and mixed oil diet compared to piglets from sows fed the hemp seed oil diet.

## Discussion

The present experiment confirmed that the fatty acid profile in sows’ colostrum and mature milk, blood plasma, and plasma of their progeny was influenced by the dietary fatty acid profile of oil sources. Previous studies revealed the impact of maternal diet provided during late gestation and lactation differing in fatty acid composition on sows’ colostrum and mature milk composition [2, 6, 12, 14, 15]. However, the present experiment is the first one using the oil obtained from hemp seeds for sow nutrition. Inclusion of hemp seed oil to the sow lactation diets was reflected in the higher proportions of 18:4n-3 (SDA) and 18:3n-6 (GLA) in colostrum and mature milk, and the elevated proportion of SDA in sows’ plasma. As SDA was not present in soybean oil, its presence in colostrum, mature milk and plasma of sows fed the 5% soybean oil may originate from metabolism of 18:3n-3. The presence of higher levels of GLA and SDA via inclusion of high levels of hemp seed oil elevated the proportions of particularly C20:4n-6 and C20:5n-3 in sow colostrum and mature milk, which is consistent with results obtained in the study by Tanghe et al. [32] who used echium oil as a source of ALA and SDA. Interestingly, plasma of sows fed hemp oil had elevated levels of C20:5n-3 but not C20:4n-6 (ARA) when compared to soybean oil-fed sows. Notably, the highest proportion of ARA in sows’ plasma was obtained on day 2 of lactation. This increase at day 2 after farrowing, and the relatively high level of ARA in sows’ plasma (but low in milk) when compared to other fatty acids may indicate the importance of this fatty acid for sows, and the change in concentration during lactation is in accordance with previous studies [8, 32]. Inclusion of hemp oil also increased plasma concentration of eicosapentaenoic acid, EPA (C22:5n-3), while no influence of dietary fat sources was obtained with regard to the concentration of docosahexaenoic acid, DHA (C22:6n-3). This result indicates lack of conversion, and probably only provision of fish oil to the maternal diet would influence the concentration of DHA in sows as shown before [2, 8, 32].

The fatty acid composition of piglets’ plasma indicated the capability of piglets to totally convert the SDA (18:4n-3) obtained from sows’ milk to further intermediates of ALA by biochemical transformation. Thus, piglets of sows fed hemp seed oil had elevated plasma concentrations of C18:3n-3, C20:5n-3 and C22:5n-3, whereas no SDA was detected in plasma of piglets. Although no significant influence of dietary treatments was obtained with regard to the concentration of DHA (C22:6n-3), its presence may indicate some conversion from EPA to DHA by the piglets, which confirmed results reported by Tanghe et al. [32]. Though piglets were provided with more GLA via the milk when sows were fed hemp seed oil rather than soybean oil, the concentration of GLA in piglets plasma was elevated when sows were fed soybean oil when compared to hemp oil, whereas the concentration of C20:3n-6 was higher in piglets receiving milk from hemp oil-fed sows. However, with regard to the products of the C20:3n-6, i.e., the C20:4n-6 and the C22:5n-6, no difference between hemp and soybean oil treatments was obtained. Thus, in accordance with Tanghe et al. [32], no benefit of providing SDA via the hemp oil could be obtained with regard to the concentration of DHA.

The inclusion of 5% hemp seed oil to the sow diets lowered the n-6/n-3 ratio in milk and plasma of sows and their progeny compared to the soybean oil-containing diets. The dietary ratio of n-6/n-3 PUFA in the present experiment ranged from 4.3:1 for the hemp seed oil diet to 9.5:1 for the soybean oil diet. Although no dietary recommendation of n-6/n-3 fatty acid ratio is available for pig nutrition [33] it should be emphasized that a high ratio of n-6/n-3 fatty acids contained in typical swine diets is a potential concern, as imbalance may limit the production of anti-inflammatory eicosanoids derived from eicosapentaenoic acid [33, 34]. Thus, the obtained lower ratio of n-6/n-3 in piglets suckling sows fed hemp oil may be considered beneficial for piglet health and immunity. In human it is well recognised that optimal n-6/n-3 ratio is important for growth and development, especially for nervous tissue and brain, which is between 2:1 and 3:1 [22]. Over the past decades, research within swine nutrition has examined the relevance of dietary n-6/n-3 ratio and vitamin E for piglet health and immunity [5, 23]. Maternal diets of varying n-6/n-3 ratios affected the antioxidant status and immune cell eicosanoid response in the progeny [23] and influenced immunoglobulins, cytokines, fatty acid composition and performance of lactating sows and suckling piglets [5]. However, most studies lack sufficient dose-response data with regard to health and immunity of piglets to form a quantitative dietary recommendation of n-6/n-3 ratio in maternal diets or milk formulas.

The present experiment furthermore showed that inclusion of 5% hemp seed oil to the sow diets enhanced the body weight of the progeny during the first week of suckling when compared to the mixed oil diet with the soybean treatment being in between. Further, the glucose concentration obtained from the piglets was affected by the dietary treatments in the same manner during the entire suckling period. Considering that the effects of dietary fats on glucose-insulin homeostasis remain uncertain [35], and that glucose in our experiment was not the fasting glucose, makes it difficult to interpret the obtained effect to hemp oil fatty acids alone or other dietary components of colostrum and mature milk. Supposedly, the obtained result may somewhat indicate that dietary provision of sows with hemp seed oil at a level of 5% can improve energy supply and the liveability of piglets during early life. However, it should be noticed that the limited number of sows in this experiment makes it difficult to draw any conclusions regarding the impact of hemp oil on pig performance. Interestingly, however, the hemp oil treatment of sows decreased the levels of IgA, IgM and IgG in sows’ colostrum and mature milk, whereas plasma concentration of immunoglobulins and haematological parameters of piglets was mostly affected by the provision of the mixed oil treatment when compared to the other dietary oil treatments. It is difficult to interpret why hemp oil inclusion at 5% differed compared to other dietary oil treatments with regard to sow performance and milk immunoglobulin concentration, while piglet immunoglobulin responses and haematological parameters were elevated by the mixed oil treatment. Results obtained in the present experiment are in the agreement with results shown by Innis et al. [36], suggesting that high dietary intake of PUFA significantly alters the concentration of red blood cell and haematocrit compared to diets low in PUFA. An elevated immunoglobulin concentration in colostrum and mature milk, with parallel-increased plasma immunoglobulin concentrations in piglets, should be interpreted as enhanced passive immune protection of suckling piglets (hence indicating enhanced immune-competence [37]). However, increased levels of specific immunoglobulins may also reflect immunological responses towards infections, i.e., IgA has major immunosuppressive mechanisms in the intestine that inhibit pro-inflammatory responses to oral antigens, which may be counterbalanced by systemic immune factors, including IgG. Recent studies have investigated the dietary n-6/n-3 fatty acids with regard to similar responses as in our study in suckling [16] and weaned piglets [38]. Dietary inclusion of fish oil or soybean oil (3.8–3.9% of diet) improved growth performance of nursing piglets by increasing milk fat output [16], and the authors furthermore concluded that fish oil consumption by sows might benefit the piglets via increasing n-3 PUFAs availability and immunoglobulins (IgM and IgG) secretion. In addition, supplementation of linseed oil, with an n-6/n-3 ratio of 4.2 (in comparison with soybean oil with an n-6/n-3 ratio of 9.8) to maternal diets, increased immunoglobulins in sows’ plasma, colostrum and milk [39]. On the other hand, Yao et al. [5] observed beneficial effects on growth and immune status of the offspring when maternal diets were having an n-6/n-3 ratio of 9:1 rather than 3:1 or 13:1. In weaners, no effect of decreasing dietary n-6/n-3 fatty acids ratio from 15:1 to 5:1 was obtained with regard to piglet performance during the entire growth phase, serum lipid profile (except cholesterol), white blood cells and lymphocytes, and IgG concentration [38]. Overall, it is not clear from the literature what is the optimal ratio of n-6/n-3 fatty acids ratio for piglet performance and immunity. With regard to reproduction (in terms of number of piglets), our obtained effect of dietary fatty acids is very difficult to explain due to the limited number of sows, and to the fact that dietary treatments started at day 108 of gestation. It should be noted that the number of piglets at weaning was not affected by dietary treatments.

Differences in the outcome of various studies regarding dietary fatty acids may in fact be attributed to parallel variations in dietary vitamin E and other antioxidants, as the membrane concentration of α-tocopherol influences oxidative stability of the fatty acids and hence their immune responses. It should be noted that piglets suckling sows fed the mixed oil diet had the lowest plasma α-tocopherol concentration although the concentration of PUFAs in plasma of piglets was highest for hemp oil and lowest in soybean oil-fed sows at all times of sampling during the suckling period. However, although sows were provided a higher dietary α-tocopherol concentration when the mixed oil was included, the higher content of γ-tocopherol in the hemp oil and content of PUFAs may have affected the plasma α-tocopherol positively in piglets suckling sows provided high levels of hemp oil.

The obtained present results further indicated that in spite of constant level of dietary α-tocopherol during the whole experiment, the concentration of sows’ plasma α-tocopherol was increased with progression of lactation stage. This observation is in agreement with obtained concentrations of PUFA in plasma of sows, hence, the increasing PUFA concentration with progressing of lactation requires higher levels of plasma α-tocopherol. The relation between dietary intake of PUFA and α-tocopherol plasma concentrations has been extensively investigated in human nutrition [40, 41] whose results indicated that high PUFA levels lead to higher plasma α-tocopherol concentrations in order to prevent oxidation of lipid-containing cellular membranes as also confirmed by the present results. The opposite pattern was observed in relation to plasma α-tocopherol of piglets, which was related to lower PUFA concentration in milk as the suckling period progressed.

Considering that one of the most investigated health effects of n-3 PUFA in human is their capability to reduce serum triglyceride levels [42], it was expected to obtain lower levels of plasma triglyceride, cholesterol and other plasma lipids in plasma of piglets of sows fed diets with increased levels of n-3 PUFA. However, in disagreement with these expectations, dietary fat sources did not differ with regard to plasma lipid levels of their progeny. In our previous study [43], inclusion of tallow (5%) in diets for weaners increased the serum concentration of cholesterol and triglycerides in comparison with sunflower oil and fish oil. Probably, the inclusion of saturated fatty acids rather than mono- and polyunsaturated fatty acids would have greater impact than the ratio of n-6/n-3 fatty acids with regard to these parameters in pigs. Our results could, however, also be connected with applied dosage of hemp seed oil, i.e., there is no evidence in the literature suggesting optimal dosage of n-3 PUFA lowering blood lipid levels, thus, supposedly, dietary n-3 levels applied in the present experiment were not optimal to achieve the effect obtained for human serum lipid levels.

As our previous studies demonstrated clear effects of dietary fatty acid composition on tissue fatty acid composition of piglets [44] including intestinal epithelial and mucosal fatty acids composition [43], we measured the expression of *COX-2, IL-10* and *TNF-α.* These genes are expected to be influenced when membrane n-3 PUFAs compete with arachidonic acid as substrates from cyclooxygenase and lipoxygenase enzymes, decreasing the production of arachidonic acid-derived eicosanoids such as prostaglandin E_2_ (PGE2), which can potentially affect immune regulation [5, 12, 45] in the piglets. While differences between intestinal segments were obtained with regard to the expression of these genes in the present study, little influence of fatty acid composition of maternal diets was obtained, which is in accordance with our additional study on inclusion of hemp cake in diets for weaned pigs [46].

## Conclusions

Our research demonstrated that colostrum and milk fatty acid composition and immunoglobulin concentration, as well as fatty acid profile of sows’ and piglets’ plasma, were influenced by the fatty acid composition of the maternal diets provided during late gestation and lactation. The hemp seed oil resulted in direct maternal supply with n-3 LCPUFAs, especially ALA and SDA, and piglets were able to convert these fatty acids obtained via the sow milk intake to C20:5n-3 and C22:5n-3. Furthermore, the obtained effect of 5% hemp seed oil with regard to piglets’ initial body weight and plasma glucose level is an interesting topic for further research, i.e., the capability of hemp seed oil to benefit piglets during early life.

## Figures and Tables

**Fig. 3:**
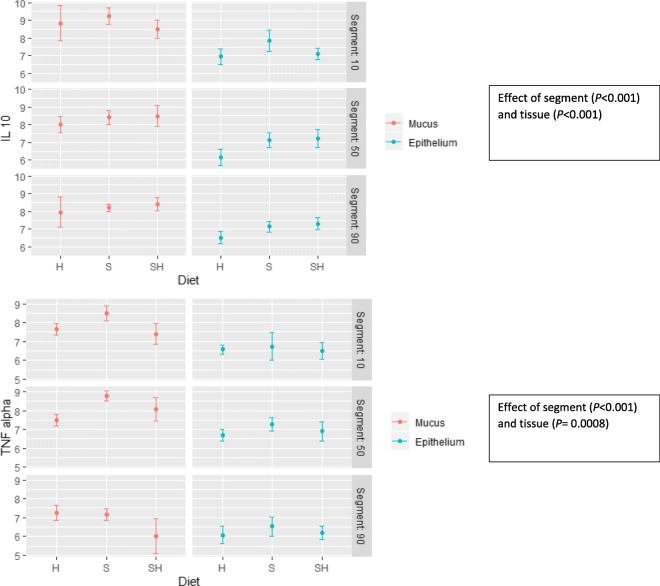
Gene expression (∆Ct) of *COX-2, IL-10, TNF-α* in piglets intestine. H = basal diet and hemp seed oil in ratio 95:5, HS = basal diet and mix of soybean and hemp seed oils (50:50) in ratio 95:5; S = basal diet and soybean oil in ratio 95:5; basal diet formulated according to Danish Nutrient Standards for Lactating Sows [24]. Segment 10 = proximal part of small intestine; segment 50 = mid part of small intestine and segment 90 = distal part of small intestine; type of tissue = mucus layer and intestinal epithelium

## Data Availability

The data sets used and analysed during the current study are available from the corresponding author on reasonable request.

## Abbreviations

ALA: α-linolenic acid
ARA: Arachidonic acid
BHB: Beta-hydroxybutyrate
COX-2: Cyclooxygenase-2
Ct: Cycle threshold
DHA: Docosahexaenoic acid
DM: Dry matter
EPA: Eicosapentaenoic acid
GAPDH: Glyceraldehyde-3-phosphate dehydrogenase
GLA: γ-linolenic acid
GLC: Gas–liquid chromatography
H: Hemp seed oil-containing diet
HS: Hemp seed and soybean oils-containing diet
IL-10: Interleukin
LCPUFA: Long-chained polyunsaturated fatty acids
MCH: The mean corpuscular haemoglobin
MCHC: The mean corpuscular haemoglobin concentration
MCV: The mean cell volume
MUFA: Monounsaturated fatty acids
NEFA: Non-esterified fatty acids
NLR: Neutrophils to lymphocytes ratio
PCR: Polymerase chain reaction
PGE2: Prostaglandin E_2_
PUFA: Polyunsaturated fatty acids
S: Soybean oil-containing diet
SDA: Stearidonic acid
SFA: Saturated fatty acids
THC: ∆9-tetrahydrocannabinol
TNF-α: Tumour necrosis factor-alpha
WBC: White blood cells
