# Correction to: Effects of dietary hemp seed oil to sows on fatty acid profiles, nutritional and immune status of piglets

**DOI:** 10.1186/s40104-020-00452-3

**Published:** 2020-05-27

**Authors:** D. Vodolazska, C. Lauridsen

**Affiliations:** Department of Animal Science, Faculty of Technical Sciences, Aarhus University, Blichers Allé 20, 8830 Tjele, Denmark

**Correction to: J Anim Sci Biotechnolo**

**https://doi.org/10.1186/s40104-020-0429-3**

In the original publication of this article [1], a figure of gene expression of Cox-2 in the Appendix 1 is missing. The correct Appendix is attached.

The original publication has been corrected.

## Figures and Tables

**Fig. 3:**
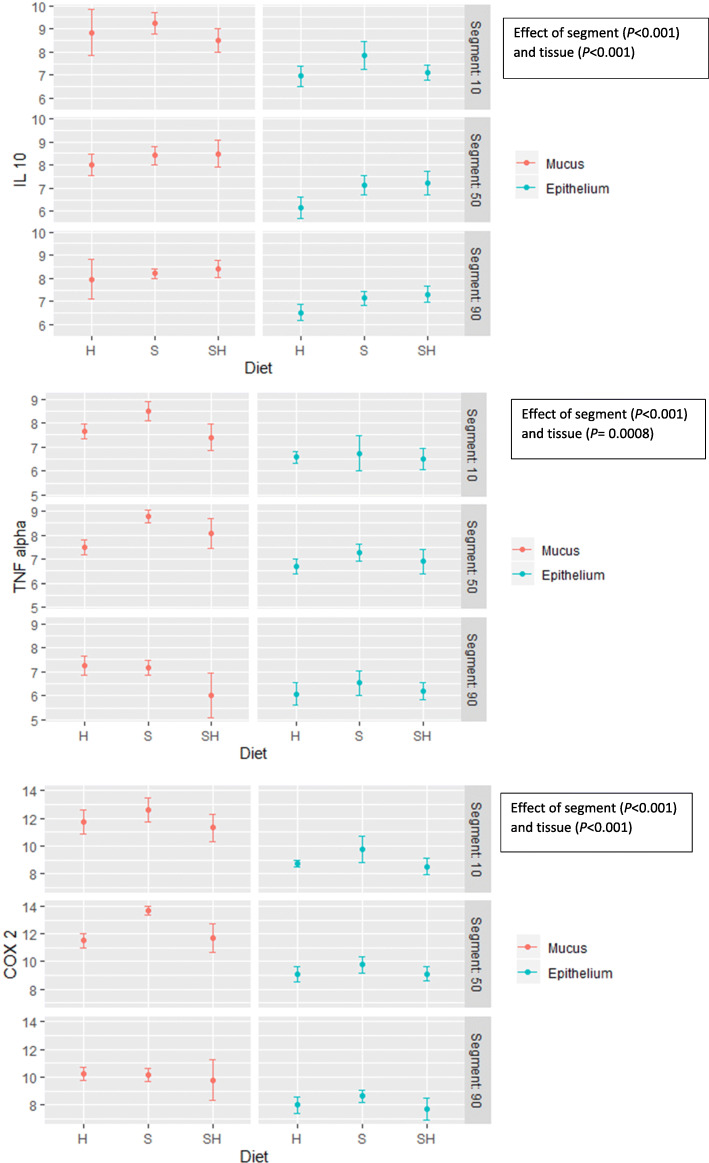
Gene expression (∆Ct) of *COX-2, IL-10, TNF-α* in piglets intestine. H=basal diet and hemp seed oil in ratio 95:5, HS= basal diet and mix of soybean and hemp seed oils (50:50) in ratio 95:5; S= basal diet and soybean oil in ratio 95:5; basal diet formulated according to Danish Nutrient Standards for Lactating Sows [24]. Segment 10 =proximal part of small intestine; segment 50= mid part of small intestine and segment 90 = distal part of small intestine; type of tissue =mucus layer and intestinal epithelium
